# 3D Aerohydrogel Scaffolds for Brain Tissue Engineering and *In Vitro* Neuroscience

**DOI:** 10.1021/cbe.5c00104

**Published:** 2026-02-20

**Authors:** Torge Hartig, Luise Schlotterose, Grace Atteh, Alexandrina Turcanu, Atharva Markale, Gabriel Chan, Margarethe Hauck, Thomas Strunskus, Ralph Lucius, Rainer Adelung, Fabian Schütt, Franz Faupel, Benjamin R. Freedman, Kirsten Hattermann, Stefan Schröder

**Affiliations:** † Chair for Multicomponent Materials, Department of Materials Science, 9179 Kiel University, Kiel 24143, Germany; ‡ Department of Orthopaedic Surgery, Beth Israel Deaconess Medical Center, 1811 Harvard Medical School, Boston, Massachusetts 02115, United States; § Institute of Anatomy, 9179 Kiel University, Kiel 24143, Germany; ∥ Department of Physiology, Anatomy and Genetics, University of Oxford, Oxford OX1 2JD, United Kingdom; ⊥ Functional Nanomaterials Chair, Department of Materials Science, 9179 Kiel University, Kiel 24143, Germany; # Kiel Nano,Surface and Interface Science, KiNSIS, Kiel University, Kiel 24143, Germany

**Keywords:** initiated chemical vapor deposition, polymers tissue engineering, biomaterials, glial cells

## Abstract

The development of 3D scaffolds addresses a critical gap in neural tissue modeling by mimicking the physiological architecture in brain cell culture. This is a key challenge to improve the reliability of *in vitro* assays and reduce animal testing, but also mandatory for the successful engineering of neural tissues in the future. Recently various types of applicable scaffolds have been applied for 3D cell cultures, which are typically porous hydrogels or fibrous mats produced by electrospinning. These scaffold materials oftentimes have a 3D structure limiting the ingrowth of cells and the diffusion of biophysical factors throughout the material. In this study, we show the capability of Aerohydrogels, fabricated via initiated chemical vapor deposition, to allow biophysical communication throughout the material in a spatially divided brain cell coculture of microglia and astrocytes. The recently developed Aerohydrogels have an ultralow density and mechanically stable 3D hollow fibrous structure. The analysis of Interleukin inflammatory pathways shows the protective influence of astrocytes within the coculture by intercellular communication. The findings support the great applicability of the newly applied Aerohydrogels in 3D brain cell coculture and relevant analysis methods like live cell imaging, cell viability assays, or gene expression. This successful establishment allows more dedicated future applications and optimization of Aerohydrogels in neural tissue modeling.

## Graphical abstract

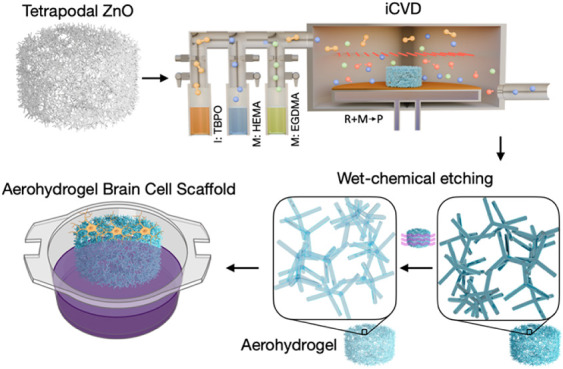

## Introduction

The central nervous system (CNS) is a complex tissue composed of diverse, highly specialized cell types organized in a three-dimensional (3D) architecture. Traditional two-dimensional (2D) cell culture systems often fail to replicate the spatial organization and dynamic cell–cell interactions that are critical to brain function. Furthermore, current animal models often struggle to predict human responses due to interspecies differences, while costs, ethical concerns, and practical concerns further limit their use.^1,2^ As a result, there is a growing need for advanced *in vitro* models that more accurately mimic the native brain microenvironment. *In vitro* test systems have the benefit of very controlled experimental conditions, fewer ethical questions, and lower consumption of resources. While they can hardly resemble every condition in a living being, they are still very important for basic research and preanimal studies. Therefore, it is important to continuously improve humanized in vitro test systems. A special focus nowadays is on 3D cell culture systems. Cells grown on regular plastic well plates display different characteristics than cells grown in a 3D setup.^3^ Hence, to mimic physiological conditions as closely as possible, different 3D cell culture systems have been developed.^4^ Traditionally scaffolds are fabricated by processes such as freeze-drying or the encapsulation of microparticles, which lead to porous unnatural adhesion sites and limitations in pore size and interconnectivity, which cause restrictions in cell movement and the transport of chemicals. Scaffolds fabricated by electrospinning resemble fibrous shapes closer to the extracellular matrix but typically lack cell infiltration.^5,6^ While these materials have a large open volume, their porous structure is not ideal for the mass transport needed for interaction with the surrounding.^7^ Scaffold materials for biological applications require appropriate diffusion of metabolites and nutrients.^8^

Recently, to address these limitations, Aerohydrogels have been developed as a new type of 3D cell scaffold.^9^ Aerohydrogels are fibrous 3D networks, resembling extracellular matrix shapes, have an ultralow density, and can be tuned chemically and mechanically based on the precursors used in the fabrication process. Aerohydrogels are created by coating a sacrificial tetrapodal ZnO (t-ZnO) scaffold (Figure 1a)^10,11^ with hydrogel thin films via initiated Chemical Vapor Deposition (iCVD)^12^ (Figure 1b). iCVD was chosen as a solution-free process that can form conformal polymer thin films on complex substrates while allowing full retention of precursor chemistry, which even allows the deposition of hydrogel thin films with tailored properties.^13–15^ For this, gas flows of vinyl-functionalized precursors and a peroxide initiator are controlled via needle valves before entering a vacuum reactor. Inside the vacuum reactor, resistively heated wires at relatively low temperatures of 200–400 °C are used to crack the peroxide initiators into radicals while keeping the monomers fully intact. Radicals with a low sticking coefficient then start a controlled radical polymerization on the cooled sample stage containing the t-ZnO scaffolds, promoting the adhesion of the monomers. This leads to a structure of hydrogel-coated interconnected microrods. The following wet-chemical etching of the ZnO creates networks of interconnected hollow hydrogel microtubes (Figure 1c). iCVD has recently been used to design biomaterial interfaces for specific cell types based on applied iCVD chemistry.^15–18^

**Figure 1:**
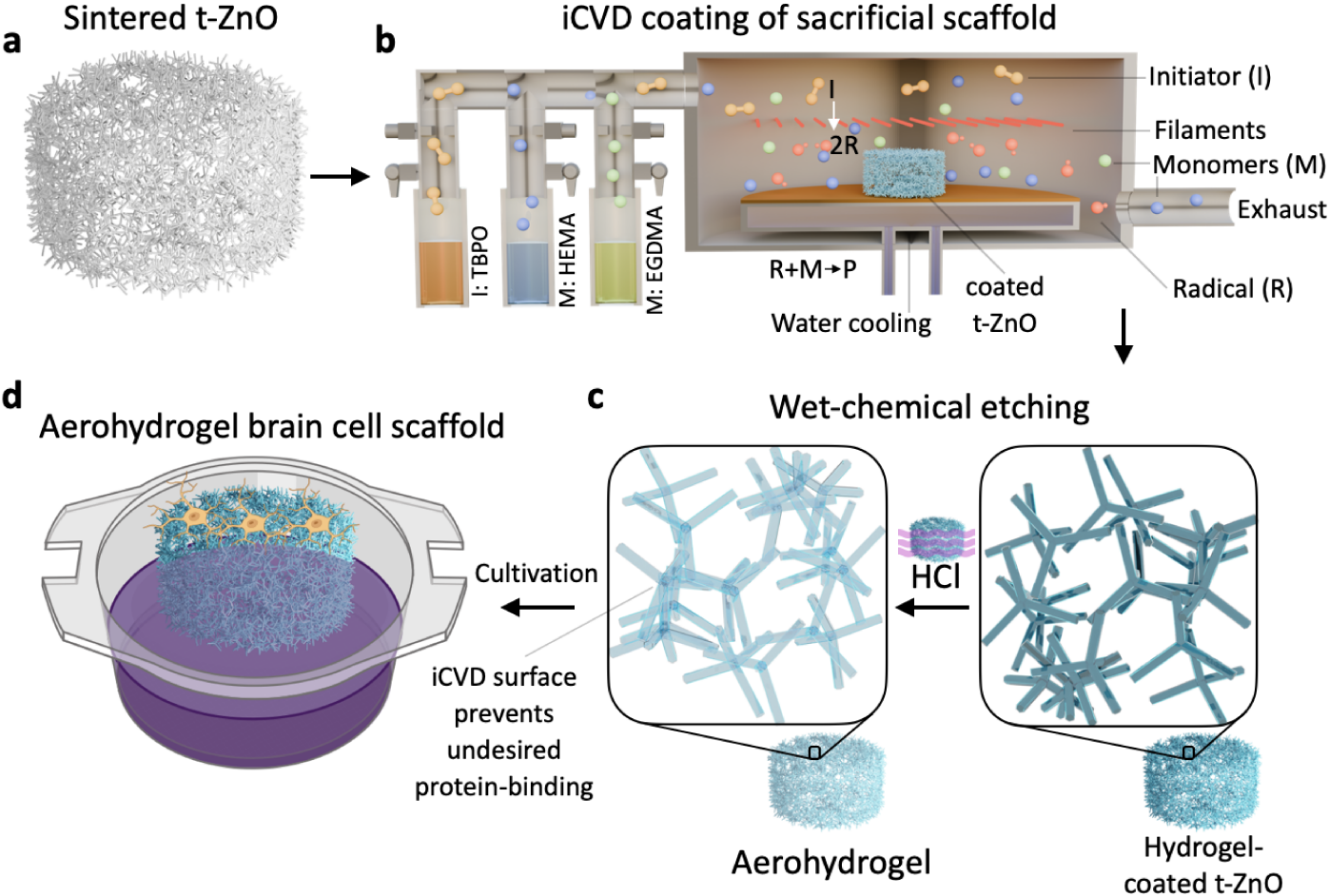
Fabrication of aerohydrogels for neural tissue modeling. **a** A sintered cylindrical t-ZnO scaffold. **b** iCVD is used to coat the t-ZnO 3D sacrificial scaffold with poly­(HEMA-*co*-EGDMA). Here, I represents an initiator molecule, M a monomer, R a radical, and P the polymer. **c** After wet-chemical etching, the Aerohydrogel cell scaffold of hollow microtubes is created. **d** Aerohydrogels were used to cultivate different brain cells.

Especially, applications of iCVD thin films in neural interfaces and brain cell culture systems have shown great success.^19–23^ Of the wide range of available iCVD polymers,^12^ pHEMA (2-hydroxyethyl methacrylate) has been reported to have the mechanical properties and resistance to prevent undesired protein binding, required in a scaffold material, while being highly biocompatible.^17,23–25^

Within this work, iCVD-based Aerohydrogels are for the first time successfully applied as cell scaffolds for different kinds of glial brain cells, representing a softer class of tissue than the previously applied C2C12 muscle cells.^9^ Furthermore, the application of Aerohydrogels in a coculture system without physical contact is examined by combining microglia and astrocytes.

## Results and Discussion

Aerohydrogels of similar composition have previously been analyzed in detail referred to as “low cross-linking Aerohydrogels”.^9^ To ensure the correct chemical composition of the materials, Fourier-transformed infrared spectroscopy (FTIR) was performed on Si-wafers placed next to the t-ZnO during the iCVD process (Figure 2a). The absorption intensity is given in arbitrary units for wavenumbers from 500 cm^–1^ to 4000 cm^–1^ in a step width of 4 cm^–1^. The hydroxyl band can be seen between 3300 cm^–1^ and 3600 cm^–1^ representing the HEMA monomer. The carbonyl band, which is present in both monomers but twice in EGDMA, is also clearly present at 1730 cm^–1^. This indicates the successful retention of the functional groups of the precursors.

**Figure 2:**
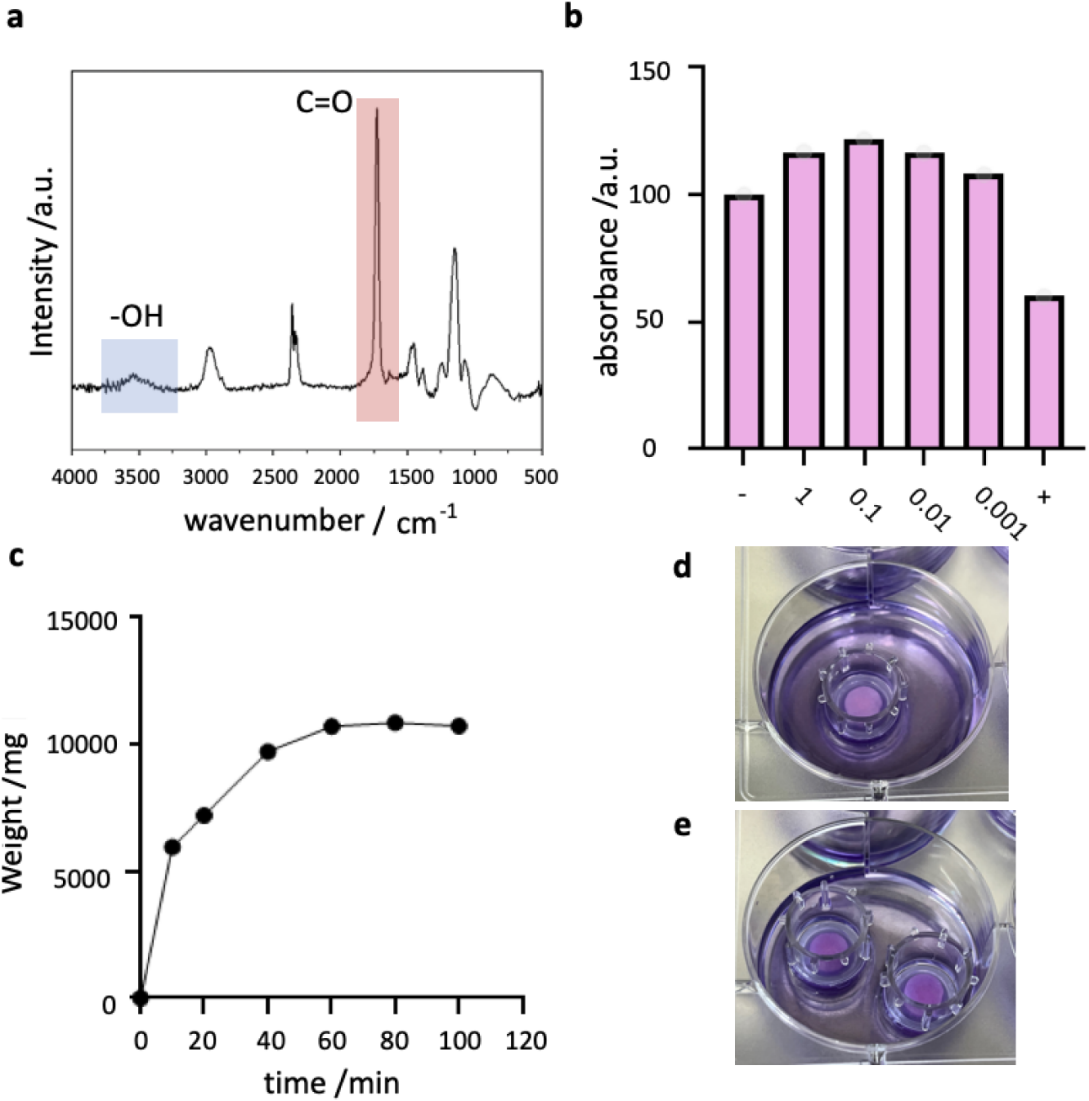
Characterization of Aerohydrogel materials. **a** FTIR spectrum of a dry iCVD coating on a silicon wafer. **b** WST measurement for cytotoxicity. Aerohydrogels were incubated in complete media and diluted 10, 100, and 1000 times, respectively. All concentrations and controls are relative to blank controls (microplate values), and the mean values (*n* = 3) were plotted, respectively. The negative control [−] contained complete media, cells, and WST-1 reagent. The positive control [+] contained 20% Dimethyl sulfoxide, cells, and WST-1 reagent. **c** Swelling of Aerohydrogel in water. **d** Representative images of a monocultured Aerohydrogel in a transwell insert. **e** Representative image of a cocultured Aerohydrogel.

Hydrogels characteristically swell in aqueous media. The swelling of the Aerohydrogels used was measured over time in water (Figure 2c). The filling with water of the free volume of the Aerohydrogel reaches a plateau after 60 min. If required, this could be accelerated by an ultrasonic bath in future applications.

The choice of iCVD-coated t-ZnO templates for Aerohydrogel fabrication can be understood in relation to three scaffold classes thoroughly tested in the literature. First, uncoated ZnO, including t-ZnO, is known to be cytotoxic when in direct contact with individual mammalian cells due to excessive Zn^2+^ release that surpasses the buffering capacity of the cells. This has been demonstrated in detail for t-ZnO by Papavlassopoulos et al.^26^ Consequently, uncoated t-ZnO networks are not suitable as biological controls for long-term neural culture. Second, inverted t-ZnO architectures embedded in hydrogels have been successfully implemented and biologically evaluated in our recent 3D-printing study with Poon and coworkers.^27^ The hydrogel matrix provides a soft, cell-friendly environment in these systems, while the remaining t-ZnO branches function as predefined guidance tunnels for neuronal outgrowth. Although these hydrogel-based or inverted-template scaffolds support neural cell viability and guided extension, they differ fundamentally from the current work: they do not allow the “free” three-dimensional growth and interconnectivity that define the iCVD-based Aerohydrogel architecture.

Third, coated ZnO microtube networks, such as CNT- or polymer-coated t-ZnO, are known to exhibit substantially higher mechanical stiffness than aeromaterials. In some cases, coatings can increase the effective modulus dramatically, reaching values in the high kPa to MPa regime.^28^ In contrast, the present study aims to achieve brain-like elastic moduli in the low-kPa range, which requires converting the coated-ZnO template into a hollow, compliant, and cytocompatible Aerohydrogel scaffold.

Due to the nanometer-thin iCVD film representing the wall thickness of the Aerohydrogel, direct μCT is not usable to analyze the porous structure as the resolution is limited. However, previous work on t-ZnO-based materials shows the inverted structure in μCT which proves interconnectivity not only of the pores between the t-ZnO structures but even of the hollow Aerohydrogel channels based on the t-ZnO structure.^29–31^ Furthermore, the porosity of these aeromaterials can be tailored through their processing parameters.^32^

The stiffness of the Aerohydrogels was characterized in a previous study to be 40.8 ± 6.5 kPa [1 Hz], being higher than native brain tissue.^9^ This higher stiffness allows handling of the samples, which can be tedious below 10 kPa.

To confirm the biocompatibility of the Aerohydrogels used and to exclude any toxicity of the iCVD polymer, a cytotoxicity assay using primary tendon-derived fibroblasts was performed. Initial and diluted conditioned media concentrations did not exhibit any toxic effects on the cells (Figure 2b). The stable cell viability indicates that the Aerohydrogel has no risk of cytotoxicity when cultured with mammalian cells. In the next step, to use the Aerohydrogels for cell scaffold applications, the Aerohydrogels were placed in 96-well plate transwell inserts to ensure an optimal supply of media. Monoculture (Figure 2d) as well as coculture setups (Figure 2e) were used with brain cells.

To investigate the applicability of the Aerohydrogels as neural tissue models, human immortalized microglia and astrocytes were used. Nowadays, these two cell types are recognized for their high importance in maintaining brain health and orchestrating neuroinflammation. Astrocytes are the most abundant cell type in the brain and are responsible for brain homeostasis and extracellular matrix (ECM) development. Their health is essential to preserve neuronal function and protect brain function.^33–35^ Therefore, they were chosen to be cultured as an exemplary brain cell type along with microglia, the resident immune cells of the CNS.^36^

Human stable cell line microglia were seeded on the Aerohydrogel scaffolds and successfully grown for multiple days. Live cell staining with calcein AM revealed a large number of living cells on the scaffold surface after 3 days in culture (Figure 3a). During this short time span, cells had already begin to immigrate into the scaffold volume (Figure 3b). Cell viability was also confirmed by a resazurin-based viability assay (Figure 3 c). This assay displayed that dye added to the media in the well plate was reduced to its fluorescent component by the cells seeded on the scaffold, in comparison to medium-only blanks (*p* < 0.05). Hence, there is a profound exchange between the medium in the well plate and the scaffold, reaching the cells seeded on the surface. Oftentimes diffusion in scaffold materials is difficult due to their respective structure. Cells inside the scaffold can suffer from a reduced supply of nutrients and oxygen.^37,38^ The Aerohydrogel scaffolds, in contrast, enable continuous diffusion through the scaffold due to their high porosity. This allows for a sufficient exchange of medium components between the media in the well plate and the scaffold. Furthermore, as shown in Figure 3d, SEM images indicate that the microglia adapted well to the porous surface of the scaffold. These results indicate that human microglia cells tolerate the scaffold material well for shorter timespans and will enable us to further investigate their beneficial properties for long-term cultivation.

**Figure 3:**
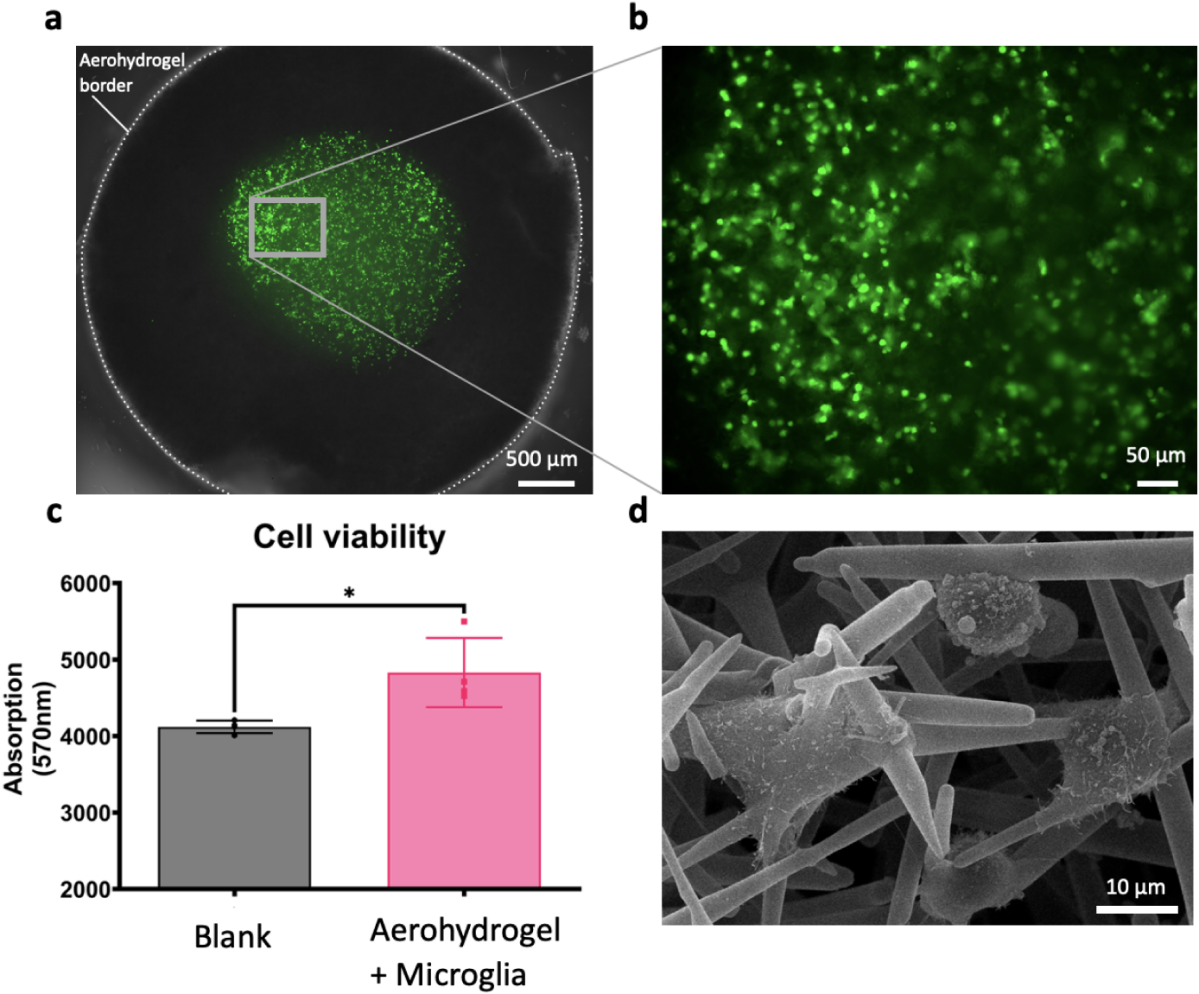
Human microglia cells grow on Aerohydrogel scaffolds. **a** Representative live cell staining (green, Calcein-AM) of human microglia grown on the Aerohydrogels for 3 days. **b** Enlargement of the relevant area on the scaffold. **c** Higher reduction of resazurin to resorufin (absorption at 570 nm) compared to a medium-only blank, displaying cellular health and exchange of compounds from the media with the cells inside the Aerohydrogel. **d** Representative scanning electron micrograph of human microglia attached to the tetrapod structure of the Aerohydrogels. *n* = 3 independent cell cultures, unpaired two-tailed Student’s *t* test with *p < 0.05.

After treatment with LPS (1 μg/mL/24 h), cells seeded in regular plastic well plates (*p* < 0.05) and microglia cells seeded on the scaffolds in monoculture (*p* < 0.01) displayed a significant reduction in cell viability compared to untreated controls. In contrast, the cell viability of microglia seeded with astrocytes on the scaffolds in coculture did not decline significantly (Figure 4a–c). Interestingly, the drop in cell viability was most profound, with a reduction of almost 50%, for microglia cells seeded on the scaffolds in monoculture. This might be due to microglia being intrinsically activated by culturing in 2D on a plastic surface. This intrinsic activation might be reduced when cultivated in a more physiologically relevant 3D environment.

**Figure 4:**
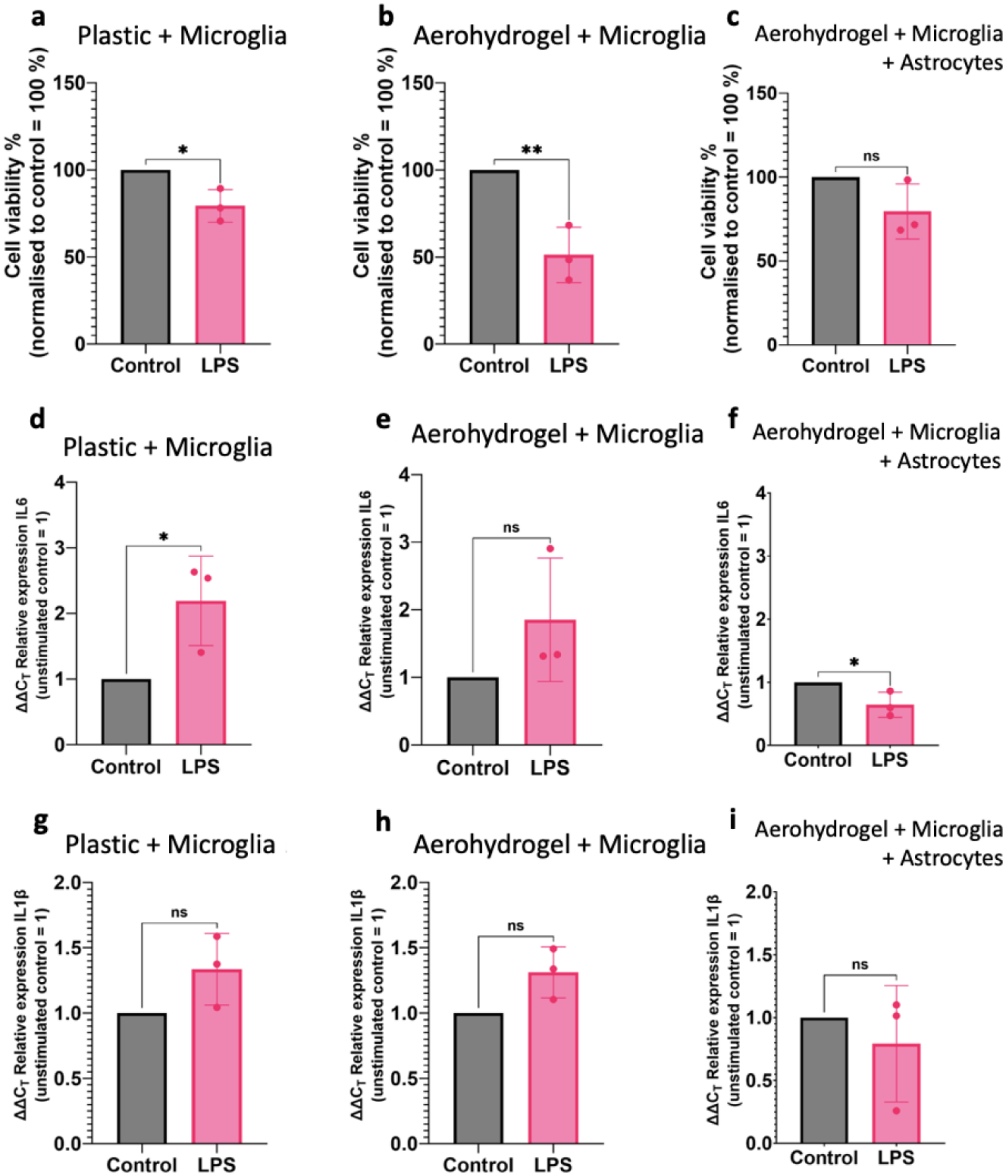
Human microglia cells and astrocytes display responsiveness to LPS treatment (1 μg/mL/24 h) in mono- and cocultures. **a**–**c** Cell viability assays of microglia mono- and microglia–astrocyte cocultures. **d**–**f** qPCR analysis of IL6 and **g–**-**i** qPCR analysis of IL1β after the LPS challenge. *n* = 3 independent cell cultures, unpaired two-tailed Student’s *t* test with *p < 0.05, **p < 0.01.

Apart from changes in cell viability, also the microglial responses at the mRNA level were analyzed by qPCR. Microglia cells as the resident immune cells in the CNS can produce high amounts of proinflammatory cytokines, which can promote harmful neuroinflammation. IL6 and IL1β are two of the most abundant proinflammatory cytokines.^39^ The addition of LPS increased the mRNA levels of IL6 for cells seeded in regular plastic well plates (*p* < 0.05) and for microglia cells seeded on the scaffolds in monoculture by almost 2-fold compared to untreated controls (Figure 4d–f). However, microglia cells that were grown with astrocytes on the scaffolds in coculture displayed in comparison to untreated controls a reduction in mRNA levels of IL6. The trend for IL1β was similar but with a lower overall response at the mRNA level (Figure 4g–i). Schmitt et al. already showed that astrocytes can have protective effects on microglia cells.^40^ These results indicate that microglia cells grown on the scaffold respond to treatment with LPS. Furthermore, this reveals the modulating influence of the astrocytes in the coculture and thereby demonstrates efficient communication between the cocultured cells without direct cell contact.

The established coculture system enables the analysis of soluble factor-mediated effects between multiple cell types that are cultured together but remain physically separated. This separation provides the advantage of simplifying downstream analysis of cellular responses, eliminating the need for time-consuming cell sorting procedures. In contrast to traditional coculture approaches such as transwell systems, the Aerohydrogel platform is not limited to two or three cell types; instead, multiple scaffolds can be cultured simultaneously within a single 3D environment, allowing detailed investigation of cellular crosstalk. These features make it possible to observe the astrocyte-mediated modulation of microglial activation in a more physiologically relevant and spatially resolved manner than conventional models. To further enhance the system, human immortalized cell lines can be replaced with human primary brain cells or cells derived from induced pluripotent stem cells.

## Conclusion

By closely mimicking the native brain microenvironment, Aerohydrogels fill a critical need in neural tissue modeling. The Aerohydrogels have successfully been applied as cell scaffolds for different brain cell types and could be quickly adapted to the requirements of other tissues. Their chemical analysis via FTIR validates the radical polymerization via iCVD as well as the retained chemical functionality. The WST indicates no cytotoxicity, and the cell viability assays of microglia and astrocytes in monoculture and coculture have also been tested successfully, showing biocompatibility and decreased intrinsic cellular reactivity. Interestingly, a protective behavior of the astrocytes to the microglia in response to LPS could be observed, highlighting that despite the cells being physically separated, crosstalk between the cells is not impaired. The Aerohydrogel structure thus allows the diffusion of biophysical cues from one scaffold to the other required for cellular communication in cocultures. While the Aerohydrogels are generally chemically stable in various solvents (Figure S1), future applications in brain tissue engineering could use the possibility of biodegradation via ester groups that should be tested.^41^ As byproducts of the degradation are undesired, the low density of the scaffolds and the low number of byproducts during the degradation make it an excellent material choice for neural tissue engineering in the future. This work lays the foundation for the application of Aerohydrogels to replace brain tissue after surgical intervention or trauma.

## Materials and Methods

### Aerohydrogel Fabrication

Tetrapodal ZnO (t-ZnO) was pressed into a cylindrical shape as a loose powder. The shape was 3 mm in diameter and 1 mm high. The pressed ZnO was then sintered for 5 h at 1150 °C to interconnect the tetrapodal network.

The interconnected tetrapodal ZnO cylinders were then coated by initiated chemical vapor deposition (iCVD) with a thin hydrogel film. Within the same chamber, witness wafers were coated for analysis of the film. The monomers used for the coating were 2-hydroxyethyl methacrylate (HEMA) (97%, stabilized with 4-methoxyphenyl; abcr) and ethylene glycol dimethacrylate (EGDMA) (98%, stabilized with 4-methoxyphenol, Alfa Aesar). As an initiator di*tert*-butyl peroxide (TBPO) (Sigma-Aldrich) was used. The reactor and the gas supply lines were heated to 100 °C. The monomers were heated to 45 °C (HEMA) and 65 °C (EGDMA). The initiator was held at room temperature. A scroll pump (nXDS 10i, Edwards, Burgess Hill, UK) was used to evacuate the reactor and gas supply system. For the nitrogen flow, a mass flow controller (MC Series, Alicat Scientific Inc., Tucson, USA) was used. Monomer and initiator flows were controlled by needle valves (Swagelok). The pressure in the reactor was controlled by a capacitive manometer (MKS Baratron) that was connected to a butterfly valve (VAT 615). A nickel-chromium filament array (Ni80/Cr20, Goodfellow GmbH) was resistively heated to 42.3 W by a power supply (Delta Elektronika, SM- 7020-D). The substrates were cooled to 30 °C with a circulating thermostat (Huber CC-K6).

Further information can be found in ref 9.

### Fourier-Transform Infrared Spectroscopy

Fourier-transform infrared spectroscopy was performed using an FTIR spectrometer (Invenio R, Bruker, Billerica, USA) in transmission mode from 4000 cm^–1^ to 500 cm^–1^. The measured data were baseline-corrected (polynomial baseline) by plotting software (Origin 2017, OriginLab, Northampton, USA).

### Swelling Measurements

The water retention of the Aerohydrogels was determined by weighing them at intervals of 0, 20, 40, 60, 80, 100, and 120 min after immersing them in deionized water at room temperature.

### Cytotoxicity Assay

Colorimetric WST-1 assay was used as a cytotoxicity evaluation for the Aerohydrogel by measuring the metabolic activity of mammalian cells in contact with the material. The formazan dye intensity was measured at an absorbance wavelength of 450 nm corresponding to metabolic activity and suggests either cell viability or apoptosis. Absorbance values for initial and diluted conditioned media concentrations were within the same range for the negative control and double that of the positive control. The increased absorbance values indicate that the Aerohydrogel has no risk for cytotoxicity when cultured with mammalian cells. The cytotoxicity assay was performed using the cell proliferation reagent WST-1 [Sigma-Aldrich, 5015944001]. To make the conditioned media, Aerohydrogel samples were added to cell culture media containing high-glucose Glutamax DMEM supplemented with pyruvate, 1% Streptomycin/Penicillin, and 10% Fetal Bovine serum [10569010, Thermo Fisher Scientific ]. The leaching process for the conditioned media occurred over 24 h. Dilutions of 1, 0.1, 0.01, and 0.001 fractions were made from the conditioned media. The negative control consisted of cell culture media, cells, and WST-1 reagent. The positive control consisted of 20% DMSO [D2650, Sigma-Aldrich], cells, and WST-1 reagent. Primary tendon-derived fibroblasts were seeded at a concentration of 5 × 10^4^ cells/well in 96-well tissue culture grade flat-bottom plates with a total volume of 100 μL of medium/conditioned medium. Cells were incubated for 24 h at 37 °C and 5% CO_2_. Ten microliters of WST-1 reagent was added to each well and incubated for 4 h at 37 °C and 5% CO_2_. After thoroughly shaking the plates for 1 min, absorbance was measured at 450 nm on a plate reader [Synergy Mx, BioTek].

### Brain Cell Culture

The human fetal astrocyte cell line SVGA was kindly provided by the group of Christine Hanssen Rinaldo, University Hospital of North Norway^42^ with the permission of Altwood.^43^ The human microglia cell line HMC3 (catalog no. CRL-3304, RRID: CVCL_II76) was purchased from the American Type Culture Collection (ATCC, Manassas, Virginia, USA). Cells were maintained in Dulbecco’s modified Eagle’s medium (DMEM) (#41965, Thermo Fisher Scientific, Darmstadt, Germany) supplemented with 10% fetal bovine serum (FBS) (#P30-3306, PAN-Biotech GmbH, Aidenbach, Germany), 1% penicillin–streptomycin (10,000 U/ml, #15140122, Thermo Fisher Scientific), 1% additional l-glutamine (#56-85-9, Carl Roth, Karlsruhe, Germany), and incubated at 5% CO_2_/37 °C. Cells were routinely checked for mycoplasma contamination by mycoplasma-specific PCR (#11-1100, Venor GeM Classic; Minerva Biolabs, Berlin, Germany).

### Experimental Cell Culture Design

Aerohydrogel scaffolds were left to soak in DMEM without FBS for 96 h. The medium for microglia cells and astrocytes was changed 96 h before cell seeding to DMEM containing only 5% FBS. For all experiments, DMEM without FBS was used. For cell seeding, the surface of the scaffolds was swabbed with filter paper and placed in cell culture inserts (#141082, Nunc, Thermo Fisher Scientific) in 6-well plates. Cells were seeded on the scaffold (40000 cells/sample) in one single drop, and 1.2 mL of DMEM without FBS was filled in the well around the inset. The medium was renewed after 48 h.

Apart from overall viability, microglia cell responsiveness to stimuli was analyzed. For Lipopolysaccharide (LPS) treatment, 1 μg/mL LPS from *Escherichia coli* O26:B6 (#L2654, Sigma-Aldrich/Merck, Taufkirchen, Germany) was added to the medium. After an additional 24 h of incubation, the scaffolds were processed as described in the respective section. Therefore, human microglial cells were treated with Lipopolysaccharide (LPS) (1 μg/mL/24 h) to evaluate their responses. Moreover, these responses were monitored in a monoculture of microglia cells and a coculture with human astrocytes. The system setup allows the placement of two or more scaffolds in one-well plate to easily create cocultures without risking the activation of major histocompatibility complex (MHC) molecules.^44^

### Cell Viability Staining

Living cells were stained using Calcein acetoxymethyl ester (Calcein-AM, no. C3099, Invitrogen, Thermo Fisher Scientific). Calcein-AM (1 μg/mL) was directly added to the medium and incubated for 20 min at 37 °C. Scaffolds were briefly rinsed with PBS and imaged using the Keyence BZx800 Fluorescence Microscope (KEYENCE GmbH, Neu-Isenburg, Germany).

### Cell Viability Assay

Cell viability was monitored using the AlamarBlue Cell Viability Reagent (#DAL1025, Thermo Fisher Scientific). AlamarBlue is a resazurin-based, membrane-permeable assay, in which resazurin reduction by living cells directly correlates with cell viability. The reagent was added to the medium (100 μL/1.2 mL), according to the manufacturer’s instructions, and incubated for 3 h. Fluorescent intensity was measured with a Tecan Infinite M Plex microplate reader and normalized to the blank and control samples.

### Quantitative Polymerase Chain Reaction (qPCR)

The scaffolds were harvested and homogenized in the TRI Reagent (#T9424, Sigma-Aldrich/Merck) using bead tubes (type E, #740815, Macherey-Nagel, Düren, Germany). The tubes were vortexed for 10 min to ensure the lysis of all cells inside the scaffolds. Subsequently, total RNA was isolated following the manufacturer’s protocol. Genomic DNA was digested using RNase-free DNase (1 U/μL, #89836, Thermo Fisher Scientific), and cDNA was synthesized with the Thermo Scientific RevertAid RT Kit (#K1691, Thermo Fisher Scientific). TaqMan primers and probes (Thermo Fisher Scientific) and HOT FIREPol Probe Universal qPCR Mix (#08-17-00001, Solis BioDyne, Tartu, Estonia) were utilized to analyze samples with the ABI PRISM 7500 sequence detection system (Applied Biosystems). The analyzed genes were GAPDH (Hs99999905_m1), IL6 (Hs00985639_m1), and IL1β (Hs01555410_m1).

The cycle threshold (CT) values were determined through the instrument software, and ΔCT values = CT­[gene of interest] – CT­[GAPDH] were calculated. Due to the logarithmic reaction mode, a ΔCT value of 3.33 corresponds to 1 order of magnitude lower gene expression compared to GAPDH. For cytokine-induced mRNA regulation, ΔΔCT values were calculated as follows: ΔΔCT = 2^–(ΔCT[stimulus]−ΔCT[control])^.

### Scanning Electron Microscopy (SEM)

Scaffolds were washed with PBS and fixed for 30 min in 3% glutaraldehyde. In the next step, the samples were washed with PBS and kept in a 2% osmium solution for 20 min. Subsequently, all water was removed by placing the samples in increasing ethanol concentrations (30–100%), and critical point drying was carried out using a CPD 030 (Bal-Tec, Balzers, Liechtenstein). Finally, the samples were coated with an SCD 050 sputter coater (Bal-Tec, Balzers, Liechtenstein) for 50 s and imaged using a JSM-IT200 SEM (JEOL, Freising, Germany).

### Statistical Analysis

All results are presented as mean values ± standard deviation (SD) and the numbers of biological replicates are stated in the respective figure legends. Statistical analysis was performed using *GraphPad Prism* V9.4.1 (RRID: SCR_002798). Statistically significant differences were assessed using a two-tailed Student’s *t* test for paired comparisons. Probabilities were considered statistically significant at values *p* < 0.05.

## Supplementary Material



## Data Availability

The data that supports the findings of this study are available from the corresponding author upon reasonable request.

## Notes

Figure S1: Stability of Aerohydrogels in organic solvents after 15 min and 14 days; solvents tested: DMSO, DCM, toluene; water as control (PDF)
