# Supplementary material for: 3D Aerohydrogel Scaffolds for Brain Tissue Engineering and In Vitro Neuroscience
Source: Chem Bio Eng. 2026 Feb 20;3(4):487–95. doi: 10.1021/cbe.5c00104 (PMC13112044; doi:10.1021/cbe.5c00104)
Supplement: Supplementary file 1 [file be5c00104_si_001.pdf]

**Supporting Information**

**3D Aerohydrogel Scaffolds for Brain Tissue Engineering and *In Vitro* Neuroscience**


Torge Hartig[a,b,#], Luise Schlotterose[c, d.#], Grace Atteh[b], Alexandrina Turcanu[a], Atharva Markale[b], Gabriel Chan[a], Margarethe Hauck[e], Thomas Strunskus[a,f], Ralph Lucius[c], Rainer Adelung[e,f], Fabian Schütt[e,f], Franz Faupel[a,f], Benjamin R. Freedman[b], Kirsten Hattermann[c], Stefan Schröder[a,f,*]

[a]Chair for Multicomponent Materials, Department of Materials Science, Kiel University, Kiel, Germany

[b]Department of Orthopaedic Surgery, Beth Israel Deaconess Medical Center, Harvard Medical School, Boston, MA, USA

[c]Institute of Anatomy, Kiel University, Kiel, Germany

[d]Department of Physiology, Anatomy and Genetics, University of Oxford, Oxford, United Kingdom

[e]Functional Nanomaterials Chair, Department of Materials Science, Kiel University, Kiel, Germany

[f]Kiel Nano, Surface and Interface Science, KiNSIS, Kiel University, Kiel, Germany

[#]equal contribution

*corresponding author: ssch@tf.uni-kiel.de

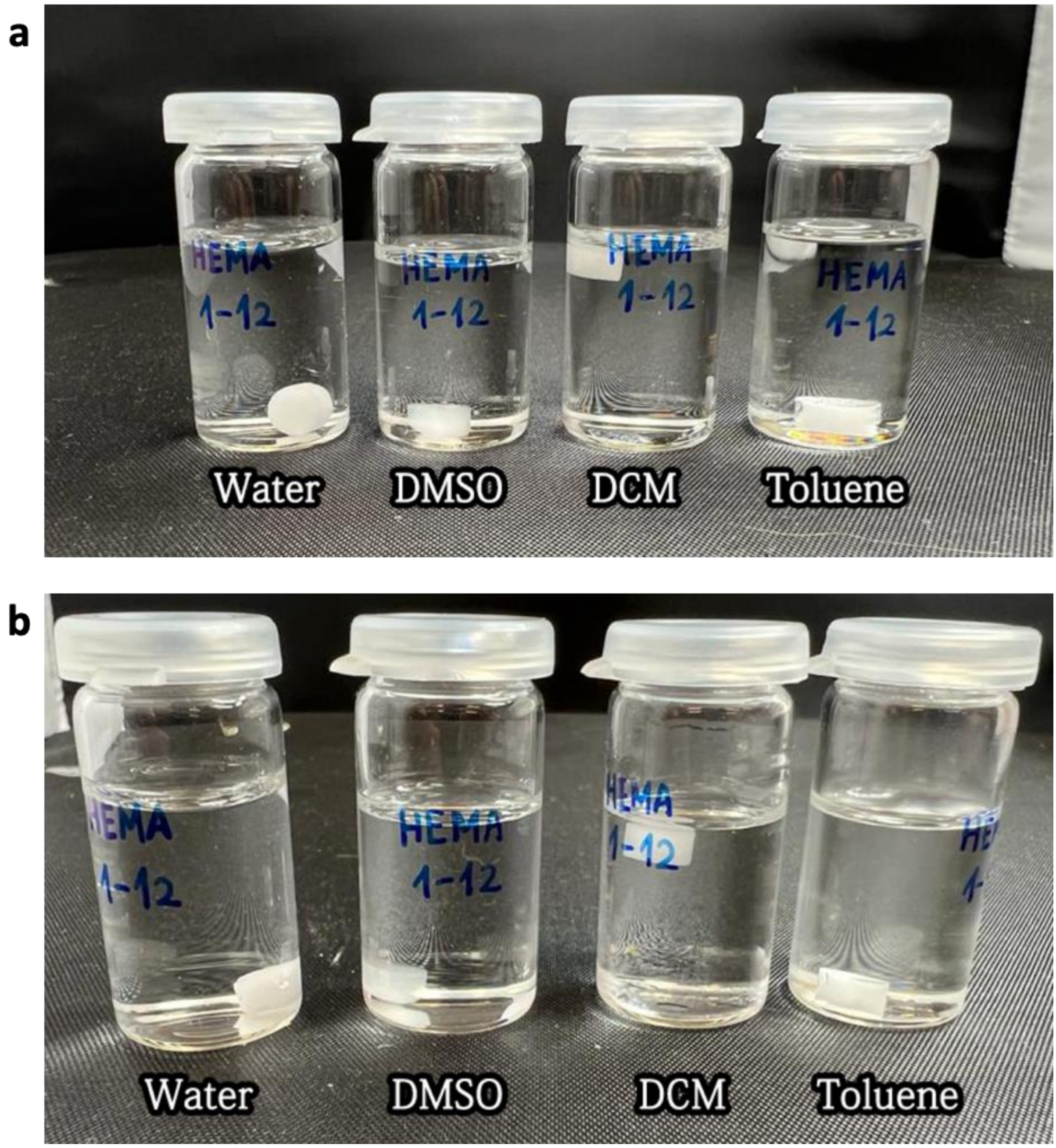


**Figure S1**. Stability of Aerohydrogels in organic solvents. **a** shows the Aerohydrogels in the solvents after 15 mins, and **b** after 14 days. Aerohydrogels were previously soaked in DI water. Solvents used are dimethyl sulphoxide (DMSO, Carl Roth GmbH, 99.5%), dichloromethane (DCM, Carl Roth GmbH, 99%) and toluene (Sigma-Aldrich, 99.5%). Water was used as a control. Aerohydrogel samples are floating in DCM probably due to immiscibility of water and DCM. All Aerohydrogels are intact after 2 weeks.
